# Unveiling Functional Impairment in Fabry Disease: The Role of Peripheral vs. Cardiac Mechanisms

**DOI:** 10.3390/biomedicines13071713

**Published:** 2025-07-14

**Authors:** Geza Halasz, Chiara Lanzillo, Raffaella Mistrulli, Emanuele Canali, Elisa Fedele, Paolo Ciacci, Federica Onorato, Guido Giacalone, Giovanni Nardecchia, Domenico Gabrielli, Federica Re

**Affiliations:** 1 Division of Cardiology, Cardio-Thoracic and Vascular Department, Azienda Ospedaliera San Camillo Forlanini, 00152 Rome, Italy; geza.halasz@gmail.com (G.H.); dgabrielli@scamilloforlanini.rm.it (D.G.); re.federica77@gmail.com (F.R.); 2 Division of Cardiology, Santo Spirito Hospital, 00193 Rome, Italy; chiaralanzillo@hotmail.com; 3 Department of Clinical and Molecular Medicine, Sapienza University of Rome, 00185 Rome, Italy; 4 Division of Cardiology, Policlinico Casilino Hospital, 00169 Rome, Italy; emanuele.canali@gmail.com (E.C.); elisafedele@hotmail.it (E.F.); 5 Campus Bio-Medico, University Of Rome, Rome 00128, Italy; pa.ciacci@gmail.com; 6 Cardiology Unit, Department of Emergency and Critical Care, Policlinico Tor Vergata, 00133 Rome, Italy; fede.onorato@gmail.com; 7 Division of Cardiology, Azienda Ospedaliera Universitaria Sant’Andrea, 00189 Rome, Italy; guido.giacalone@uniroma1.it; 8 Faculty of Medicine and Surgery, Sapienza University of Rome, 00185 Rome, Italy; giovanni13.nardecchia@gmail.com

**Keywords:** Fabry, cardiopulmonary exercise testing, exercise capacity

## Abstract

**Background:** Anderson–Fabry disease (AFD) is a progressive lysosomal storage disorder characterized by systemic glycosphingolipid accumulation. While cardiac imaging plays a central role in disease monitoring, the relationship between structural myocardial changes and exercise capacity remains incompletely defined. This study aimed to evaluate functional impairment in AFD patients using cardiopulmonary exercise testing (CPET) and to determine whether limitations are primarily cardiac or extracardiac in origin. **Methods:** Thirty-one patients with genetically confirmed AFD were retrospectively enrolled from two tertiary centers. All underwent baseline clinical assessment, resting transthoracic echocardiography (TTE), spirometry, and symptom-limited CPET using a cycle ergometer and a 10 W/min ramp protocol. Echocardiographic parameters included the LVEF, global longitudinal strain (GLS), E/e′ ratio, TAPSE, and PASP. CPET measurements included the peak VO_2_, anaerobic threshold (AT), VE/VCO_2_ slope, oxygen pulse (VO_2_/HR), and VO_2_/watt ratio. **Results:** The mean age was 48.4 ± 17.6 years, with most patients classified as NYHA I. LVEF was preserved (62.3 ± 8.6%), and diastolic indices were within normal limits (E/e′ 7.1 ± 2.4), but GLS was impaired (11.3 ± 10.5%). CPET showed reduced peak VO_2_ (18.6 ± 6.1 mL/kg/min; 71.4% predicted) and early AT (40.8%), with preserved ventilatory efficiency and oxygen pulse. VO_2_/watt was mildly reduced, suggesting peripheral limitations despite intact central hemodynamics. **Conclusions:** Functional impairment is common in AFD patients, even with mild cardiac involvement. CPET reveals early systemic limitations not captured by standard imaging, supporting its role in phenotypic characterization and therapeutic decision-making.

## 1. Introduction

Anderson–Fabry disease (AFD) is a rare X-linked lysosomal storage disorder caused by α-galactosidase A deficiency, leading to progressive glycosphingolipid accumulation—particularly globotriaosylceramide (Gb3)—within lysosomes across multiple organ systems including the heart, kidneys, nervous system, and skin [1]. Cardiac involvement is a major driver of morbidity and mortality in AFD and typically manifests as left ventricular hypertrophy (LVH), arrhythmias, and heart failure (HF), particularly as the disease progresses [2].

Standard transthoracic echocardiography (TTE) plays a central role in the cardiac assessment of AFD, allowing the evaluation of left ventricular wall thickness, chamber dimensions, and systolic and diastolic function and the estimation of pulmonary pressures. In the early stages of AFD, echocardiographic findings may be entirely normal, while more advanced stages typically demonstrate concentric LV hypertrophy, impaired relaxation, increased left atrial volume, and, eventually, overt systolic dysfunction [3]. However, subtle myocardial dysfunction may be present before overt structural abnormalities become detectable on standard TTE. Advanced imaging techniques such as global longitudinal strain (GLS) and cardiac magnetic resonance (CMR) tissue characterization (T1 and T2 mapping) have increasingly revealed subclinical myocardial involvement, even in asymptomatic or early-stage patients [4,5].

Early diagnosis and the timely initiation of disease-specific therapy—such as enzyme replacement therapy (ERT) or chaperone therapy—are crucial, as the therapeutic efficacy appears greater when treatment is started before irreversible organ damage occurs [6]. Therefore, identifying early functional markers of systemic involvement is critical in optimizing patient outcomes.

Although cardiac imaging provides valuable structural information, the impact of AFD on exercise tolerance and overall functional capacity remains incompletely characterized. Cardiopulmonary exercise testing (CPET) offers a unique integrative evaluation of cardiovascular, pulmonary, and peripheral performance during physical effort, potentially revealing functional impairment before overt structural damage becomes apparent [7,8]. Despite this potential, the role of CPET in the routine evaluation of Fabry patients has not been fully established.

The predefined hypothesis of this retrospective observational study was that functional impairment, detectable by CPET, may occur even in the presence of mild or minimal structural cardiac involvement on standard TTE, reflecting systemic or subclinical mechanisms that precede overt cardiomyopathy. Specifically, we aimed to clarify whether the degree of exercise intolerance observed in AFD patients correlates with measurable cardiac structural abnormalities or whether extracardiac factors may contribute significantly even in the early stages of the disease.

## 2. Materials and Methods

### 2.1. Patient Selection and Study Protocol

We retrospectively analyzed 31 consecutive patients with genetically confirmed AFD from two specialized centers in Rome, Italy (San Camillo Hospital and Policlinico Casilino), between 2020 and 2024. AFD diagnosis was confirmed by GLA gene mutation analysis, and 71% of patients exhibited a phenotype-positive presentation based on clinical and imaging findings. All patients underwent a comprehensive baseline evaluation including clinical history, physical examination, laboratory testing, and the assessment of renal function. Functional status was classified according to the New York Heart Association (NYHA) scale.

All patients underwent resting transthoracic echocardiography (TTE), performed by experienced sonographers and interpreted by certified cardiologists according to ASE/EACVI guidelines.

Global longitudinal strain (GLS) was measured using speckle-tracking echocardiography from standard apical views (four-, two-, and long-axis). Images were acquired with an adequate frame rate (60–90 fps) and analyzed offline using vendor-independent software (TomTec Imaging Systems, Unterschleißheim, Germany). GLS was calculated as the average peak systolic strain across all 17 left ventricular segments.

- Two-dimensional imaging was used to assess left ventricular (LV) morphology and function, including measurements of interventricular septal and posterior wall thickness, maximal wall thickness, LV end-diastolic diameter, and global GLS where feasible.
- Doppler evaluation included the pulsed-wave Doppler assessment of mitral inflow (E and A waves) and tissue Doppler imaging (TDI) of the mitral annulus to calculate the E/e′ ratio, an estimate of the LV filling pressure. Continuous-wave Doppler was used to estimate the tricuspid regurgitant velocity and derive the pulmonary artery systolic pressure (PASP).
- M-mode echocardiography was utilized to measure tricuspid annular plane systolic excursion (TAPSE) as an index of right ventricular systolic function.

For all patients, CPET was performed at the time of their first comprehensive evaluation at our center, typically within one month of genetic diagnosis confirmation and, where applicable, within one month from the initiation of enzyme replacement therapy. Before CPET, spirometry was performed to evaluate ventilatory function and exclude significant pulmonary limitations. The parameters measured included the forced vital capacity (FVC), forced expiratory volume in one second (FEV_1_), and maximum voluntary ventilation (MVV). CPET was conducted on an upright cycle ergometer using a 10 W/min ramp protocol. Gas exchange was continuously analyzed using a calibrated breath-by-breath system with a mouthpiece-mounted sensor. The test was considered valid if the patient achieved a respiratory exchange ratio (RER) > 1.05. Patients failing to meet this criterion were excluded. The parameters recorded included the peak oxygen consumption (pVO_2_), ventilatory efficiency (VE/VCO_2_ slope), oxygen pulse (VO_2_/heart rate), anaerobic threshold (AT), breathing reserve (BR), and end-tidal CO_2_ pressure (PETCO_2_). Heart rate, blood pressure, and perceived exertion were monitored throughout the test. All CPET and echocardiographic data were digitally recorded and reviewed offline for consistency and accuracy.

### 2.2. Statistical Analysis

Continuous variables are expressed as the mean ± standard deviation (SD) or median and interquartile range (IQR), depending on the data distribution, which was assessed using the Shapiro–Wilk test. Categorical variables are reported as counts and percentages. Statistical analyses were conducted using R software (version 4.3.1; R Foundation for Statistical Computing, Vienna, Austria).

## 3. Results

The study population included 31 patients with genetically confirmed Anderson–Fabry disease (AFD). The mean age was 48.36 ± 17.63 years, with 36% males. The majority of patients were asymptomatic (NYHA class I: 80.96%) and had preserved renal function (eGFR 85.85 ± 31.83 mL/min/1.73 m^2^).

The genetic analysis identified multiple GLA gene variants. The most frequent mutations were c.644A > G (p.Asn215Ser) (n = 14, 45%), followed by c.730G > A (p.Asp244Asn) (n = 7, 23%), c.337T > C (p.Phe113Leu) (n = 1), c.160C > T (p.Leu54Phe) (n = 1), c.1079G > A (p.Gly360Asp) (n = 3), and c.680G > T (p.R227L) (n = 1). Based on genotype–phenotype associations, the majority of mutations were classified as late-onset variants, while only a minority were compatible with classic AFD.

Regarding ongoing treatment, 39% (12 patients) were on beta-blockers, 48% (15 patients) were on ACE inhibitors or angiotensin receptor blockers (ACEi/ARBs), and 29% (9 patients) were on combination therapy. Enzyme replacement therapy (ERT) was ongoing in 19 patients (61.3%) at the time of evaluation.

### 3.1. Echocardiographic Parameters

**Figure 1:**
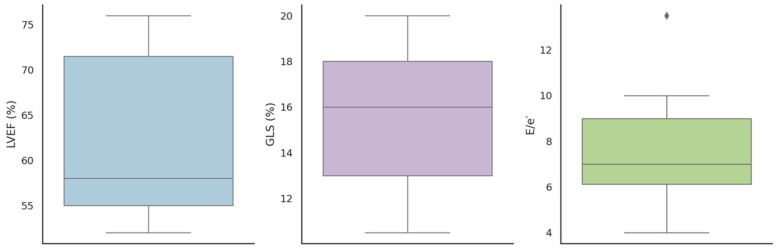
Boxplots of resting echocardiographic parameters in Fabry patients: left ventricular ejection fraction (LVEF), global longitudinal strain (GLS), and E/e′ ratio. Each plot displays the median, interquartile range, and outliers (rhombus).

At baseline echocardiography, mild septal hypertrophy was observed (interventricular septum thickness: 12.00 ± 3.65 mm; maximum wall thickness: 16.5 ± 4.4 mm). Left ventricular ejection fraction (LVEF) was preserved (62.25 ± 8.61%), and diastolic filling pressures were normal (E/e′: 7.12 ± 2.43). However, GLS was impaired (11.3 ± 10.49%), suggesting subclinical myocardial dysfunction. The left atrial volume index (LAVI) was 28.24 ± 11.83 mL/m^2^, right ventricular systolic function was preserved (TAPSE: 19.53 ± 5.60 mm), and pulmonary pressures were normal (PASP: 25.77 ± 10.25 mmHg) (Table 1, Figure 1).

### 3.2. Cardiopulmonary Exercise Testing (CPET) Results

Despite mild structural cardiac involvement, CPET revealed a consistent reduction in functional capacity. The mean peak oxygen consumption (pVO_2_) was 18.56 ± 6.11 mL/kg/min, corresponding to 71.38 ± 18.92% of the predicted value. The AT occurred early, at 40.75% of predicted pVO_2_, consistent with an early transition to anaerobic metabolism.

Ventilatory efficiency remained within normal limits (VE/VCO_2_ slope: 30.61 ± 9.09; VE/VCO_2_ at AT: 28.40 ± 2.77), as did oxygen pulse (VO_2_/HR: 10.61 ± 3.62 mL/beat) and breathing reserve (51.48 ± 11.52%), and the VO_2_/watt ratio showed only a mild reduction (9.86 ± 1.29 mL/min/W).

**Figure 2:**
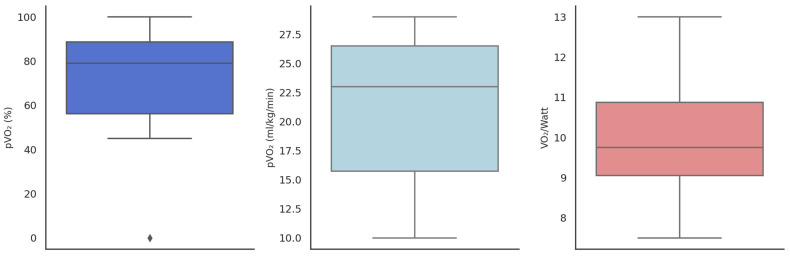
Boxplots show pVO_2_ (% predicted), absolute pVO_2_ (mL/kg/min), and VO_2_/Watt, with medians, interquartile ranges, and outliers (rhombus).

All patients achieved maximal or near-maximal effort (mean respiratory exchange ratio: 1.10 ± 0.086). The end-tidal CO_2_ pressure was within the normal range both at rest (36.97 ± 16.19 mmHg) and at peak exercise (40.95 ± 16.20 mmHg), indicating preserved ventilatory–perfusion matching (Table 2, Figure 2).

## 4. Discussion

The findings from our cohort of AFD patients highlight a crucial observation: a reduced exercise capacity is prevalent even among individuals with minimal or no overt cardiac involvement, suggesting a multifactorial etiology that extends beyond myocardial dysfunction. Despite preserved LVEF, normal pulmonary pressures at rest, and relatively mild myocardial hypertrophy, a significant proportion of patients exhibited impaired pVO_2_ and a low AT—well before the appearance of advanced cardiomyopathic features. The genetic distribution is also consistent with the overall mild-to-moderate cardiac involvement observed in the cohort.

These results are aligned with previous studies reporting reduced pṾO_2_ in AFD patients, even in early disease stages or in the absence of overt cardiac involvement [8]. One study demonstrated improvement after ERT in a small cohort of 15 patients, where exercise intolerance improved following ERT despite there being no evidence of structural cardiac progression [9]. Powell et al. further confirmed diminished cardiopulmonary fitness across various stages of disease severity [10].

Symptoms such as breathlessness, fatigue, and reduced exercise tolerance are frequently reported in Fabry disease. However, given the multisystemic nature of the disease, attributing these symptoms to a single mechanism remains challenging [11]. The normal VE/VCO_2_ slope and VO_2_/HR observed in our cohort argue against ventilatory inefficiency or impaired chronotropic response as primary causes of limitation. Instead, the combination of mildly reduced VO_2_/watt and preserved central hemodynamics supports a predominant peripheral mechanism [12].

Glycosphingolipid accumulation in the vascular endothelium may reduce nitric oxide availability, impairing vasodilatory responses and limiting oxygen delivery during exercise. In addition to endothelial dysfunction, direct skeletal muscle involvement—including Gb3 deposition, mitochondrial impairment, and autonomic dysregulation—may further compromise muscular oxygen utilization [13,14]. These hypotheses are supported by previous observations of reduced muscular efficiency and early AT, even in phenotype-negative individuals.

Other systemic contributors may include physical deconditioning, subclinical pulmonary involvement, and skeletal muscle metabolic alterations [15,16]. In addition, increasing evidence highlights the potential role of central mechanisms, including neuropsychological and neurological dysfunction. Depression and anxiety are highly prevalent in AFD patients, with some studies reporting depression rates as high as 46%, particularly among females [17]. Depression has been associated with chronic pain, poor quality of life, fatigue, and reduced motivation to engage in physical activity, potentially exacerbating exercise intolerance [18]. Furthermore, small fiber neuropathy and autonomic dysfunction—including hypohidrosis, gastrointestinal dysmotility, and blunted heart rate responses—may contribute to the limitation in exercise capacity [19]. Neurological involvement, such as cerebral small vessel disease, transient ischemic attacks, and cognitive impairment, has also been documented in AFD and may affect both motor function and participation in physical activity [20]. Although these central and neuropsychological aspects were not specifically investigated in our cohort, their potential contribution to functional limitation must be acknowledged.

In this context, the role of physical activity and exercise emerges as an important, though still underexplored, adjunctive therapeutic strategy in Fabry disease. Despite initial concerns related to potential cardiac or neurological risks, preliminary studies suggest that supervised exercise programs may improve physical function, muscle strength, aerobic capacity, and overall well-being in FD patients [21]. Exercise may also have beneficial effects on mood and quality of life, mitigating some of the psychosocial burden of the disease. This multifaceted impairment underscores the need for a comprehensive, multidisciplinary approach to the management of Fabry disease [22].

Our findings indicate that functional impairment, as assessed by CPET, is frequently present even in AFD patients with minimal or mild cardiac structural involvement. While advanced structural myocardial changes may contribute to a further reduction in exercise capacity at the later stages, functional limitation often precedes overt structural abnormalities. These observations are consistent with recent reports, such as that by Powell et al. [10], which demonstrated that there were already significant reductions in VO_2_ peak in the early phases of Fabry cardiomyopathy. This dissociation suggests that CPET may detect signs of early systemic dysfunction—including microvascular, skeletal muscular, autonomic and neuropsychological contributors—that are not fully captured by standard imaging modalities. Accordingly, CPET provides incremental and complementary information, which is particularly valuable in the early disease phases to guide clinical management and therapeutic decisions.

In addition to extracardiac contributors, our findings of functional impairment in patients with minimal or absent structural cardiac involvement on standard echocardiography are consistent with accumulating evidence from advanced cardiac imaging studies. Cardiac magnetic resonance (CMR) tissue characterization has extensively demonstrated that subclinical myocardial involvement—including glycosphingolipid accumulation (native T1 mapping), myocardial inflammation (T2 mapping), microvascular dysfunction (perfusion imaging), and early fibrosis—may precede the development of overt hypertrophy or diastolic dysfunction [14]. Importantly, a recent study by Roy et al. [14] directly demonstrated that an impaired VO2 peak was significantly correlated with early myocardial abnormalities detected by CMR. These correlations suggest that functional impairment measured by CPET may serve as an early integrative marker of subclinical cardiac involvement in Fabry disease, complementary to advanced tissue characterization provided by CMR.

Moreover, in our cohort, although left ventricular ejection fraction was preserved (62.2 ± 8.6%) and diastolic filling pressures were within normal limits (E/e′ 7.1 ± 2.4), a significant reduction in GLS (11.3 ± 10.5%) was observed. This pattern is increasingly recognized as an early indicator of subclinical myocardial involvement in Fabry disease, reflecting the myocardial storage of glycosphingolipids and subtle deformation abnormalities preceding overt hypertrophy or functional impairment based on conventional measures [22].

Recent studies integrating echocardiographic strain imaging into functional assessments have demonstrated that reduced GLS may parallel an impaired exercise capacity even in the early stages, supporting the concept that deformation imaging may capture the myocardial substrate underlying functional limitation observed during CPET [11]. However, in our cohort, although GLS was reduced in many patients despite preserved LVEF, no clear correlation was observed between GLS and peak VO_2_.

The therapeutic implications extend beyond cardiac-targeted therapies. While imaging remains essential in diagnosis and follow-up, CPET offers complementary insights into functional reserve and systemic disease burden. Recognizing extracardiac limitations may inform tailored interventions, including structured exercise programs, endothelial-targeting strategies, and anti-inflammatory or antioxidant therapies [23]. Furthermore, early CPET abnormalities could signal the need for therapeutic escalation, even in the absence of clear structural markers of cardiac disease. This supports a shift toward proactive, rather than reactive, management in Fabry cardiomyopathy [24].

## 5. Limitations

This study is limited by its cross-sectional design. Only baseline CPET data, collected at the time of initial evaluation and shortly after diagnosis (and early after ERT initiation, when applicable), were analyzed. Therefore, no longitudinal assessment of functional changes or treatment effects was possible. Furthermore, the relatively small sample size, although comparable to existing studies in this rare disease population, may limit the generalizability. Additionally, the female predominance in our cohort could have contributed to the relatively mild cardiac structural involvement observed, as hemizygous males generally present with more advanced disease due to complete enzyme deficiency. Finally, while standard echocardiographic measures were obtained, we acknowledge that more advanced imaging techniques such as CMR tissue characterization may provide additional insights into subclinical myocardial involvement.

## 6. Conclusions

In conclusion, our findings emphasize the systemic nature and clinical complexity of AFD. Even in early stages, patients may experience significant functional limitations not fully captured by imaging or clinical scores. CPET may serve as a valuable tool for early phenotypic characterization and individualized management, aiming ultimately to improve functional capacity and quality of life in this population.

## Figures and Tables

**Table 1:** Echocardiographic parameters in AFD patients.

Echocardiographic Parameters	N = 31
LVEF, %	62.25 ± 8.61
LVEDD, mm	48.00 ± 1.82
LVMi (g/m^2^)	129.6 ± 47.2
IVS, mm	12.00 ± 3.65
PWT, mm	12.25± 3.86
Max thickness, mm	16.5± 4.4
GLS, %	11.3± 10.49
E/e mean	7.12 ± 2.43
LAVi, mL/cmq	28.24 ± 11.83
TAPSE, mm	19.53 ± 5.60
sPAP, mmHg	25.77 ± 10.25

IVS, interventricular septum; GLS, global longitudinal strain; LAVI; left atrial volume index; LV EDD, left ventricular end-diastolic diameter; LVMi; left ventricular mass index; LVEF, left ventricular ejection fraction; LAVi, left atrial volume index; sPAP, systolic pulmonary artery pressure; TAPSE; tricuspid annulus plane systolic excursion; PWT, posterior wall thickness. E/e, early transmitral flow velocity to early diastolic mitral annular velocity ratio.

**Table 2:** Cardiopulmonary exercise test parameters.

CPET Parameters	N = 31
pVO_2_, mL/kg/min	18.56 ± 6.11
pVO_2_, %	71.38 ± 18.92
SBP rest, mmHg	122 ±13
DBP rest, mmHg	74 ± 9
SBP peak, mmHg	151 ± 11
DBP peak, mmHg	71 ± 8
RER	1.10 ± 0.086
AT, %	40.75 ± 14.37
VE/VCO_2_ slope	30.61 ± 9.09
VE/VCO_2_ AT	28.40 ± 2.77
PETCO_2_ rest, mmHg	36.97 ± 16.19
PET CO_2_ peak, mmHg	40.95 ± 16.20
BR, %	51.48 ± 11.52
VO_2_/HR, mL/beat	10.61 ± 3.62
VO_2_/watt	9.86 ± 1.29
APMHR	77% ± 11

pVO_2_, peak oxygen consumption; pVO_2_ (%), peak oxygen consumption as percentage of predicted value; SBP, systolic blood pressure; DBP, diastolic blood pressure; RER, respiratory exchange ratio; AT, anaerobic threshold; VE/VCO_2_, slope of the ventilatory equivalents to carbon dioxide production relation; PETCO_2_, pressure end-tidal carbon dioxide; BR, breathing reserve; VO_2_/HR, oxygen pulse; APMHR, age-predicted maximal heart rate. VO_2_/watt, oxygen consumption per unit of work rate (VO_2_ per watt).

## Data Availability

Anonymous and limited data can be made available on request. The data underlying this article will be shared upon reasonable request to the corresponding author.
